# Microbiota transplant therapy in inflammatory bowel disease: advances and mechanistic insights

**DOI:** 10.1080/19490976.2025.2477255

**Published:** 2025-03-10

**Authors:** Daphne Moutsoglou, Pavithra Ramakrishnan, Byron P. Vaughn

**Affiliations:** a Gastroenterology Section, Minneapolis VA Health Care System, Minneapolis, MN, USA; b Department of Medicine, University of Minnesota, Minneapolis, MN, USA; c Division of Gastroenterology, Hepatology, and Nutrition, University of Minnesota, Minneapolis, MN, USA

**Keywords:** Fecal microbiota transplantation, microbiota transplant therapy, ulcerative colitis, crohn’s disease, inflammatory bowel disease, microbiome

## Abstract

Microbiota transplant therapy is an emerging therapy for inflammatory bowel disease, but factors influencing its efficacy and mechanism remain poorly understood. In this narrative review, we outline key elements affecting therapeutic outcomes, including donor factors (such as age and patient relationship), recipient factors, control selection, and elements impacting engraftment and its correlation with clinical response. We also examine potential mechanisms through inflammatory bowel disease trials, focusing on the interplay between the microbiota, host, and immune system. Finally, we briefly explore potential future directions for microbiota transplant therapy and promising emerging treatments.

## Introduction

Fecal microbiota transplantation, also known as microbiota transplant therapy (MTT), is the transfer of microbiota from the stool of one donor to a recipient to treat human disease. The concept of *microbiota* was first conceptualized by the Roman scholar Marcus Terentius Varro in 30 B.C. when he wrote about small creatures that could not be visualized but could cause disease; however, the first MTTs, referred to as *yellow soup*, were performed during the fourth century in China to treat gastrointestinal illnesses.^1^ This ancient form of MTT preceded the 17^th^ century discovery of microscopic life, *animalcules*,^2^ described by Antoine van Leeuwenhoek. The first modern MTT was performed in 1958 to treat patients with pseudomembranous enterocolitis.^3^ Since then, scientists and physicians have made great strides in understanding how MTT may treat a variety of diseases. And although it has been around for more than one and a half millennia, its mechanisms remain elusive. This review summarizes the factors that may contribute to clinical response following MTT in inflammatory bowel disease (IBD) patients. We prefer the term *MTT*, rather than fecal microbiota transplant, to account for the transfer of microbiota, which as this therapy evolves may not involve the entire fecal microbiome and, in the future, may not be directly harvested from stool.

## Host response to microbiota transplant therapy in inflammatory bowel disease

### Clinical outcomes for ulcerative colitis

A Cochrane review published in 2023^4^ evaluated the efficacy of MTT for ulcerative colitis (UC) and Crohn’s disease (CD) based on its ability to induce and maintain clinical remission and endoscopic remission. For induction of clinical remission in UC, 468 participants across 10 randomized controlled trials (RCTs) were evaluated over 6–12 weeks; MTT had a relative risk (RR) of 1.79 (95% CI 1.13–2.84), although the certainty of evidence was low due to small individual trial size and heterogeneity.^4^ Five RCTs including 285 participants evaluated MTT for induction of endoscopic remission in UC between 8 and 12 weeks, finding an RR of 1.45 (95% CI 0.64–3.29) favoring MTT with low certainty of evidence.^4^ Individuals who received MTT were less likely to have adverse events (0.56, 95% CI 0.28–1.14), although with a very low certainty of evidence.^4^ Less data exists for maintenance of remission, and only two RCTs including 71 participants were evaluated. Over 48–56 weeks, MTT had an RR of 2.97 (95% CI 0.26–34.42) versus placebo for maintenance of remission in UC.^4^ The RR for any adverse events for MTT in maintenance of remission of UC was 1.16 (95% CI 0.85–1.59), favoring the control group, with very low quality of evidence; they were unable to estimate the risk for serious adverse events.^4^

Heterogeneity in definitions of disease activity, clinical, and endoscopic response limits an accurate assessment of the true effect of MTT in UC. Continued high quality, larger studies are needed to determine a consistent, accurate effect.

### Clinical outcomes for Crohn’s disease

When the 2023 Cochrane review^4^ was published, no randomized, double-blinded, placebo-controlled trials were available evaluating the induction of clinical or endoscopic remission nor the maintenance of endoscopic remission for CD with MTT. One single-blinded, randomized, multicenter trial by Sokol et al. evaluated MTT in ileocolonic or colonic CD in participants who had recently reached clinical remission (defined as a Harvey-Bradshaw Index <5) from a flare treated by oral corticosteroids.^5^ Though the primary outcome was microbial engraftment, individuals who received MTT trended toward improved steroid-free clinical remission rates (RR of 1.21; 95% CI 0.36–4.14).^4,5^ Additionally, at 24 weeks there was a non-significant, numerically lower rate of clinical disease exacerbation in the MTT group. At the time of publication of this review, no randomized, double-blinded, placebo-controlled trials for MTT in CD have been published.

### Host mucosal response following microbiota transplant therapy

Changes in the mucosal transcriptome in UC mucosal biopsies in MTT participants were analyzed from a single trial.^6^ Following MTT, shotgun sequencing of UC mucosal biopsies identified upregulated mucosal host genes involved with focal adhesion, actin cytoskeletal regulation, and tight junctions.^7^ Downregulation of genes encoding interferon regulatory 4 (*IRF4*) and guanylate-binding protein 5 (*GBP5*) were associated with remission.^7^ Murine guanylate-binding proteins mediate inflammasome activation to intracellular pathogens,^8^ and the genetic deletion of *GBP5* yields mice that are more resistant to developing colitis.^7^ *IRF4* regulates several immune functions, including T-helper 17 cell commitment,^9^ T regulatory cell differentiation,^10^ and migration of CD4^+^ T cells to the intestine.^11^ Interestingly, analysis of these genes in mucosal biopsies did not show any relevant differences between responders and non-responders at baseline, indicating that MTT induced these changes.

### Changes in immune cell populations with microbiota transplant therapy

Several trials have also evaluated MTT’s effect on immune cell populations. Oral, lyophilized MTT in UC participants reduced peripheral blood populations of mucosal invariant T cells (CD4^+^TCRαβ^+^MR1^+^),^12^ innate-like T cells that are associated with inflamed mucosa in UC participants.^13^ In another study, following a single MTT administration for CD participants, colonic effector T regulatory cells (CD4^+^CD25^+^CD127^lo^), but not CD4^+^CD39^+^CD161^+^ effector T cells (associated with Th17 cell differentiation)^14^ measured from mucosal biopsies increased relative to baseline, although this change was not associated with clinical response to MTT.^15^ It is not clear if these changes are MTT-specific, or the result of a general decrease in inflammation.

Costello et al. investigated colonic lamina propria populations in UC participants following MTT and found a significant and positive correlation of baseline B cell (CD19^+^CD20^+^CD45RO^−^) and dendritic cell (Lineage-HLA-DR^+^CD33^+^CD11c^+^) populations with baseline total Mayo scores and a negative significant correlation of natural killer (CD19/CD20^−^ CD16/CD56^+^) cells with baseline total Mayo scores.^16^ However, MTT did not significantly change these lamina propria cell populations.^16^

## Donor characteristics and selection for microbiota transplant therapy

As there is a variation of normal microbiomes, donor selection targeted to restore what is missing in IBD patients may improve clinical outcomes. Potential selection criteria have included the presence or absence of specific taxa,^17^ high diversity,^18^ or metabolic features such as high stool butyrate levels.^12^ One study noted that donor *Bacteroides* was associated with steroid-free remission while donor *Streptococcus* species was associated with non-response to MTT.^19^ Another trial selected donors based on bacterial loads (bacteria per gram of stool) yet found no difference from autologous MTT compared to healthy donor MTT in achieving the primary endpoint of steroid-free clinical remission (total Mayo ≤2, no sub-score >2).^20^ Selecting donors based on taxa known to produce short-chain fatty acids (SCFAs) did not result in consistent recipient effects.^12^ Although donor selection is appealing, at this point there are insufficient and inconsistent data to identify an optimal donor. It may be that a given donor-selection should be paired with the recipient microbiome deficiency, and future study is needed in this area.

### Should donors be genetically related to recipients?

Closer genetic relatives share more similar microbiota,^21^ potentially related to similar host genetics among relatives.^21,22^ Analysis of UC participants undergoing MTT by Ishikawa et al.^23^ on the degree of donor-relatedness supports that recipients of donors that share more genetics and are closer to each other in age have improved clinical outcomes.^24^ Of note, siblings had the highest cumulative non-relapse rate, while parent and child donors had the lowest rate, and spouse and cousin donors were intermediate between the two groups.^24^

### Super-donor: myth or phenomenon?

The term *super-donor* describes a donor that either achieves significantly more engraftment or clinical response than other donors in the same trial. Clinical data in UC trials are mixed with some trials finding a super-donor effect^25,26^ and others seeing a possible super-donor effect.^6^ To investigate this phenomenon, Olesen et al. performed a meta-analysis of sequenced samples reexamining multiple trials and showed that the available data failed to statistically demonstrate a super-donor effect.^27^ Another meta-analysis of metagenomic data from 316 MTT trials for multiple clinical indications (including IBD) supported the notion that current data do not support the super-donor hypothesis and found that recipient effects are far more important than donor effects in impacting strain-level outcomes to MTT.^28^ This study highlighted that donor-recipient compatibility drives strain turnover and donor colonization.^28^

### What controls should be used in microbiota transplant therapy trials?

A variety of controls have been used in MTT trials. Please see Table 1 for a list of blinded RCTs and a description of the control group. These include using a medication control (mesalamine enema),^18^ and inert placebo controls^12,17,29^ including saline^34^ or water^25^ enemas. Several MTT IBD trials have used autologous (the patient’s own) stool^16,20,30,33^ as a control, and only one of these trials has found a significant difference between healthy donor versus autologous stool for steroid-free remission rates (total Mayo ≤2 and endoscopic Mayo ≤1 at week 8).^16^ In this trial, healthy donor stool was prepared under anaerobic conditions and pooled; while autologous stool was not pooled and was prepared under aerobic conditions.^16^ These differences in preparation may confound results. Another trial prepared both the autologous and healthy donor stool under anaerobic conditions; however, this trial was stopped early due to futility with no difference between the groups achieving steroid-free clinical remission (total Mayo ≤2, no sub-score >1).^20^ This raises the concern that autologous stool may not be an ideal control because it is not inert. While autologous stool is useful to help with blinding in cases where MTT is delivered in a visible manner to either the patient or trial team (e.g. enema or colonoscopy), its administration could still have immunogenic properties. Further study on this topic is needed to determine if autologous MTT could have host effects similar to healthy, donor-derived MTT.

**Table 1.** Similarities and differences between single- and double-blinded randomized controlled trials of microbiota transplant therapy in Crohn’s disease and ulcerative colitis based on study design, primary outcome, route, number of doses, donor characteristics and processing factors, control group, and results of the primary outcome. An outline of engraftment assessment and results are also highlighted.

Study Design	Primary Outcome	Route	MTT Doses	Donor, Storage, Production Conditions	Control	Primary Outcome Results	Study
**Crohn’s Disease Blinded Randomized Control Trials**							
Multicenter, single-blinded, RCT	Engraftment of donor microbiota at wk 6 (Sorenson index > 0.6)	Colonoscopy	2	Unrelated, single donor per patient, fresh source^††^	Physiologic serum delivered via colonoscopy	No significant engraftment of donor microbiota^¶^	Sokol 2020^5^
**Ulcerative Colitis Blinded Randomized Control Trials**							
Single-center, double-blinded, RCT	Clinical remission (SCCAI ≤2) + ≥1-pt improvement on combined eMayo score of sigmoid + rectum vs baseline at wk 12	Nasoduodenal x1, followed by a 2^nd^ dose 3 wks later	2	Mixture of related and unrelated donors, single donor per patient^**,‡‡^	Autologous donor stool given by nasoduodenal tube	No significant difference in primary outcome between healthy and autologous donor groups^†‡^	Rossen 2015^30^
Single-center, double-blinded, RCT	Clinical remission (tMayo score < 3, eMayo = 0) at wk 7	Enema x1 per wk for 6 wks	6	Unrelated, single donor, fresh and frozen sources^‡‡^	Water enema	Significantly more remission in MTT versus control group^†¶^	Moayyedi 2015^25^
Multicenter, double-blinded, RCT	Steroid-free clinical remission and endoscopic remission or response (tMayo ≤2, all subscores ≤ 1, and ≥ 1-pt reduction in endoscopy subscore) at wk 8	Colonoscopy, then enema 5× per wk for a total of 8 wks	41	Unrelated, pooled donors (3–7), frozen source^‡‡^	Saline colonoscopy and saline enemas	Significantly more achieved primary outcome in MTT group^§^	Paramsothy 2017^6^
Multicenter, double-blinded, RCT	Steroid-free remission (tMayo ≤2 and eMayo ≤ 1) at wk 8	Colonoscopy followed by 2 enemas within a wk	3	Unrelated, pooled donors (3–4 per patient), frozen source, anaerobically prepared	Un-pooled, autologous donor, aerobically prepared	Significantly more achieved primary outcome in MTT group^§^	Costello 2019^16^
Single-center, double-blinded, RCT	Maintenance of steroid-free clinical remission (Mayo ≤2, subscores ≤ 1) at wk 48	Colonoscopy every 8 weeks	7	Unrelated donor, fresh and frozen sources^‡‡^	Sham placebo colonoscopy (saline + coloring)	No significant difference in primary endpoint^§^	Sood 2019^31^
Single-center, double-blinded, RCT	Adverse events measured up until wk 36	Colonoscopy then encapsulated, daily for 12 wks	85	Unrelated, single donor for colonoscopy. Two alternating donors for and capsule MTT. Frozen source^‡‡^	Sham colonoscopy and capsules	No significant difference in adverse events between groups^#^	Crothers 2021^12^
Multicenter, double-blinded, RCT	Corticosteroid-free clinical remission + endoscopic remission or response (tMayo ≤2, all Mayo subscores ≤ 1, and ≥ 1 pt ↓ eMayo subscore from baseline endoscopy) all at wk 8	Encapsulated (3× daily for 1 wk, then 2× daily for 1 wk, then 1× daily for 6 wks)	77	Unrelated, single donor, freeze-dried^‡‡^	Identical placebo capsules	Significantly more patients in MTT achieved primary outcome than placebo^§^	Haifer 2022^17^
Multicenter, single-blinded, RCT	Clinical steroid-free remission (SCCAI score < 3) at wk 8 between Groups 1 and 2	Two groups received colonoscopy + enemas on days 2 and 14; Group 1: MTT colonoscopy and enemas without any dietary conditioning Group 2: donor-diet conditioned MTT + patient UC exclusion diet for 12 weeks	3	Single donor*, frozen source^††^	Group 3: no MTT with just UC exclusion diet	No significant differences in remission between groups^†§^	Sarbagili Shabat 2022^32^
Multicenter, double-blinded, placebo-controlled, RCT	Maintenance of remission, fecal calprotectin <200 µg/mL, clinical Mayo score < 3 out to 12 months	Colonoscopy	1	Single donor per patient*, frozen source^‡‡^	Autologous donor stool	No significant difference between autologous and healthy donor MTT^§^	Lahtinen 2023^33^
Multicenter, double-blinded, RCT	Steroid-free clinical remission (tMayo ≤2, no sub-score >1) wk 8	First MTT via sigmoidoscopy followed by 3 enemas	4	Single donor per patient*, frozen source, strict anaerobic conditions	Autologous donor stool, strict anaerobic conditions	No significant difference^†§^	Caenepeel 2024^20^

Definition of abbreviations: eMayo= endoscopic Mayo; MTT=microbiota transplant therapy; pt=point; RCT=randomized controlled trial; SCCAI=simple clinical colitis activity index; tMayo=total Mayo score; UC=ulcerative colitis; UCEIS=ulcerative colitis endoscopic index of severity; wk=week; wks=weeks.

*Unknown relationship between donor and recipient.

^†^Trial stopped early due to futility.

^‡^Engraftment or donor similarity and clinical improvement significantly and positively correlated.

^§^Engraftment or donor similarity not assessed in study.

^¶^Engraftment or donor similarity and clinical improvement not significantly (*p* < 0.05) correlated.

^#^Engraftment or donor similarity not assessed in those with clinical improvement.

**Fresh versus frozen source of material not specified.

^††^Collected in an anaerobic container and further preparation conditions not specified.

^‡‡^Aerobic versus anaerobic conditions during preparation not specified.

## Microbiota transplant therapy processing and administration

There is no standardized method for MTT processing and administration that ensures maximal clinical benefit for IBD. Table 1 compares single- and double-blinded RCTs based on differences in processing and administration, and Table 2 provides a high-level overview of the various donor stool processing and administration routes used with a discussion on their impacts.

**Table 2.** Overview of stool processing methods, administration routes, and dosing of microbiota transplant therapy.

Microbiota Transplant Therapy Processing Factors	Notes	Study
**Donor Stool Processing**		
Aerobic	Reduction in viable species capable of producing anti-inflammatory metabolites such as short-chainfatty acids. Reduced viability and observedtaxa.^35^	Papanicolas 2019^35^
Anaerobic	Increase in alpha diversity of donor material.^16,36^ Increases obligate anaerobes^16,36^ that may have short-chain fatty acid capacity.^16^	**Costello 2019**^16^ Bernard 2023^36^ **Caenepeel 2024**^20^
**Donor**		
Single-Donor Material	May be the only option in some countries due to regulatory requirements. Allows for identification of a particular donor or preparation lot in the case of concern for transfer of infectious agents. If few donors are used in a single trial, there is a risk of skewing data toward donor-specific effects.	**Rossen 2015**^30^ **Moayyedi 2015**^25^ **Sokol 2020**^5^ **Fang 2021**^37^ **Březina 2021**^18^ **Haifer 2022**^17^ **Sarbagili Shabat 2022**^32^ **Lahtinen 2023**^33^ **Caenepeel 2024**^20^ **Moutsoglou 2024**^29^
Pooled, Multiple-Donor Material	Increases MTT bacterial diversity of donor material.^38^ Multi-donor MTT may be more effective at achieving clinical response than single donor.^38^ Another study found no difference in clinical and endoscopic remission between single versus pooled donor MTT in UC.^39^	**Paramsothy 2017**^6^ **Costello 2019**^16^ **Kedia 2022**^40^ Levast 2023^38^ El Hage Chehade 2023^39^
Un-Pooled, Multiple-Donor	Multiple donors could complement each other taxonomically and metagenomically; keeping donors separate allows for tracing infection source	**Crothers 2021**^12^
**Storage**		
Fresh	Increased alpha and beta diversity of donor material.^41^ Fresh is not superior compared to frozen when comparing symptom improvement rates in recurrent *Clostridiodes difficile* infection.^42,43^ No significant difference in remission rates^39^ or safety^42^ between fresh or frozen donor material in UC.	Lee 2016^42^ Ishikawa 2017^23^ **Paramsothy 2017**^6^ **Costello 2019** ^16^ **Sokol 2020** ^5^ **Fang** 2021^37^ **Kedia 2022** ^40^ Bilinski 2022^41^ Gangwani 2023^43^ El Hage Chehade 2023^39^
Frozen	Increased accessibility and practicality.^42^ MTT material stored for two years still contained culturable organisms but no longer showed a significant difference between samples prepared with and without the presence of atmospheric oxygen.^36^ Four-fold reduction in living bacterial cells, reduction in beta diversity.^41^ No difference in viability with freezing; however, metagenomic potential may be temporarily affected.^44^	Takahashi 2019^44^ **Crothers 2021** ^12^ **Březina 2021** ^18^ **Pai 2021** ^34,45^ **Haifer 2022** ^17^ **Sarbagili Shabat 2022** ^32^ **Lahtinen 2023** ^33^ Bernard 2023^36^ **Caenepeel 2024** ^20^ **Moutsoglou 2024** ^29^
**Delivery Route**		
Oral Encapsulated	Convenient delivery method that likely improves compliance for longer duration dosing. Gastric acid exposure might limit efficacy if formulation for encapsulation is not acid-resistant.	**Haifer 2022**^17^ **Moutsoglou 2024**^29^
Nasogastric	Possible aspiration risk, and the acidic stomach environment risks loss of viability.	Suskind 2015^46^
Nasoduodenal	Bypasses acidic stomach. Possible aspiration risk.	**Rossen 2015**^30^
Colonoscopic	Burden of procedure and risk of bowel perforation. Inconvenient if repeat treatments are needed. Allows for mucosal pre-treatment mucosal inspection.^47^	Kelly and Allegretti 2017^47^ **Sood 2019** ^48^ **Sokol 2020** ^5^ **Fang 2021** ^37^ **Kedia 2022** ^40^ **Lahtinen 2023** ^33^
Enema	Inexpensive equipment, less invasive. Less able to get donor material beyond sigmoid colon.	**Moayyedi 2015**^25^ **Schierová 2020**^49^ **Březina 2021**^18^ **Pai 2021**^45^
Transendoscopic tubing (colonic and mid-gut)	May deliver material to the entire colon. Convenient for repeat administration. Needs colonoscopy or endoscopy for placement	Wang 2023^50^ Lin 2024^51^ Zheng 2024^52^ Zhang 2024^53^
Combination of Above Methods	May integrate different mechanisms of action that different routes exert.	**Paramsothy 2017**^6^ **Costello 2019**^16^ **Crothers 2021**^12^ **Sarbagili Shabat 2022**^32^ **Caenepeel 2024**^20^
**Duration of Treatment**		
Single Session	Ease of administration	Suskind 2015^46^ Ishikawa 2017^23^ **Fang 2021** ^37^ **Lahtinen 2023** ^33^
Multiple Sessions	A systematic review and metaanalysis published on case series, prospective cohort studies, and RCTs found that repeated sessions are associated with improved pooled response and remission rates in both UC and CD versus a single session.^54^ A systematic review and metaanalysis published on RCTs in UC found no significant difference in intensive (more than once weekly) versus non-intensive (not more than once weekly) MTT administrations in clinical and endoscopic remission rates.^39^	**Rossen 2015**^30^ **Moayyedi 2015**^25^ **Paramsothy 2017**^6^ **Costello 2019**^16^ **Sood 2019**^48^ **Sokol 2020**^5^ **Crothers 2021**^12^ **Březina 2021**^18^ **Pai 2021**^34,45^ Mocanu 2021^54^ **Haifer 2022**^17^ **Sarbagili Shabat 2022**^32^ **Kedia 2022**^40^ El Hage Chehade 2023^39^ **Caenepeel 2024**^20^

Definition of abbreviations: CD= Crohn’s disease; MTT=microbiota transplant therapy.

RCT=randomized controlled trial; UC=ulcerative colitis.

Randomized controlled trials with microbiota transplant therapy in inflammatory bowel disease are bolded.

While some studies have found significant improvement in the MTT group versus placebo that hint at possible factors at play, such as anaerobically prepared healthy donor MTT in the Costello et al. trial,^16^ and pooling of donors in the Paramsothy et al. trial,^6^ no head-to-head clinical trials exist studying these. The number of bacteria present^20^ and viability^29^ in the donor stool can be impacted by processing. And different routes of administration (such as colonoscopy) have practical implications, particularly for repeat administration. Route may also impact the immune response elicited to MTT (depending on if it is particularly delivered to the small bowel versus the colon). This concept was demonstrated in a murine study where the bacterial enzyme, beta-hexosaminidase (an enzyme that is conserved across commensals, but particularly within the Bacteroidetes phylum) drove the development of small intestinal intraepithelial lymphocytes that work with peripheral T regulatory cells to suppress colonic inflammation in a murine model of colitis.^55^

Despite no direct comparative trials, most evidence for how these factors may impact outcomes have come from systematic reviews and meta-analyses of recurrent *Clostridioides difficile* infection (rCDI), IBD clinical trial literature, and clinical laboratory investigation of microbiota under different conditions.

### Aerobic versus anaerobic preparation

Most trials do not specify processing donor microbiota under anaerobic conditions; the only two that do are those by Costello et al.^16^ and Caenepeel et al.^20^ Trials using aerobic versus anaerobic preparation methods and potential effects are outlined in Tables 1 and 2. *In vitro* studies of microbiota processed in aerobic conditions show a reduction in species capable of producing anti-inflammatory metabolites such as SCFAs^35^ and an increase in alpha diversity in donor material when produced under anaerobic conditions.^36^ Despite the anaerobic conditions used in the Costello et al. trial,^16^ no significant differences in SCFAs were observed in the stools of patients in the treatment versus placebo group, and stool SCFA levels were not correlated to any treatment effects.^16^ No direct comparisons of aerobic versus anaerobic donor processing conditions exist at this time, and no analysis per a systematic review and metaanalysis exists, due to the paucity of data.

### Single-donor versus un-pooled, multi-donor versus pooled, multi-donor material

Several trials specify giving patients material from one single donor for the duration of the trial, versus others that give material from a single donor at one time point but include multiple donors during the course of the trial (un-pooled, multiple donors),^12^ and others that pool multiple different donors for a given administration, known as pooled, multiple-donor material (see Tables 1 and 2 for a list of trials using these methods and discussion of these different factors). In some countries, pooling multiple donor material is prohibited, such as by the Food and Drug Administration in the United States. One systematic review and meta-analysis published in 2023 that included data from UC RCTs, case control, and case studies found that multi-donor material was superior to single-donor material in inducing clinical remission.^38^ A second systematic review and metaanalysis published in 2023 that focused on only UC RCTs found no significant difference between combined clinical and endoscopic remission rates in those given material from single versus pooled donors.^39^ Using material from multiple donors may compliment a more full spectrum of both the taxonomy and functional potential of the microbiome and allows for better standardization of MTT but may limit source-tracing for infection or serious adverse events.

### Fresh versus frozen donor material

Donor material can either be banked and frozen, improving feasibility and ease, or may be processed immediately and given to the patient (within several hours or the same day as the donation, see Tables 1 and 2). Benefits of processing the donation immediately include, in theory, increased viability and stability of available taxa and community diversity^56^; however, several studies show that the functionality^56^ is not significantly affected by freezing, and that fresh donor material is not superior to frozen material for improving rates of rCDI.^42,43^ A systematic review and meta-analysis of UC trials found no differences in remission rates for UC between those receiving fresh versus frozen donor material.^39^

### Delivery route

Several delivery routes have been used including oral administration using capsules, targeting the upper/middle gut using nasogastric or nasoduodenal tubes, and targeting the lower gut via enema or colonoscopy, or a combination of routes (Tables 1 and 2). No single-study, direct comparisons have been pursued in IBD. Several studies of direct comparisons in rCDI have found no difference in efficacy or non-inferiority when comparing various methods of delivery (oral encapsulated versus colonoscopy^57,58^ or encapsulated versus enema).^59^ Two systematic reviews and metanalyses published in 2022 and 2023 evaluating clinical trials of MTT versus placebo in UC found no significant difference between MTT delivery to the upper versus lower GI tract for remission rates.^39,60^

### Single versus repeated administrations

Another factor includes the number of MTT administrations. Most trials give repeated dosing (see Tables 1 and 2). Thus far, no trials have compared one versus multiple doses of MTT. Theoretically, repeat MTT administrations could increase the likelihood of taxonomic changes or change the metagenomic potential to be more similar to the donor’s; however, one systematic review and metaanalysis published in 2023 of six RCTs did not find any differences in intensive (MTT delivery occurring more than once weekly) regimens versus less intensive (MTT given not more than once weekly) delivery frequencies on combined clinical and endoscopic remission rates in UC.^39^

## Donor microbial engraftment in inflammatory bowel disease

**Figure 1.**
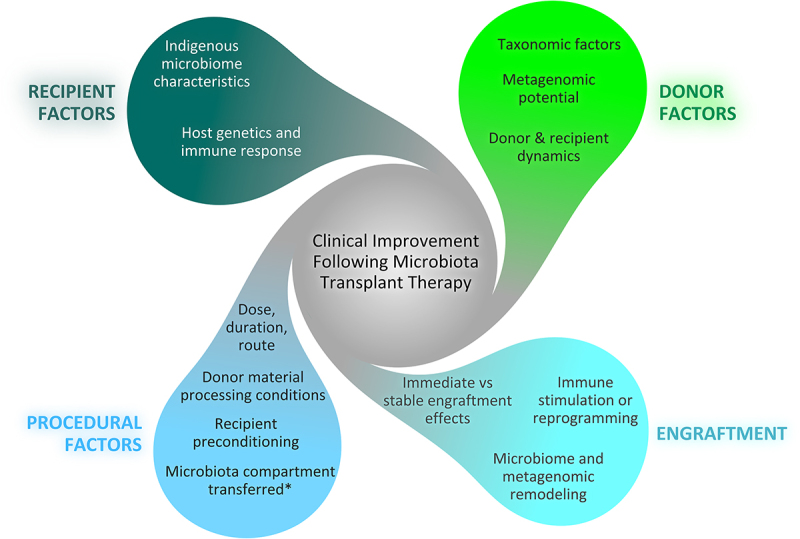
Potential challenges and mechanistic factors impacting clinical efficacy of microbiota transplant therapy. These include recipient and donor factors (which may interact dynamically), procedural factors, and the possible effects of engraftment immunologically and metagenomically.

One of the goals of donor stool processing and MTT administration is to maximize the chance to engraft donor microbiota. Additional characteristics between donors and recipients that may affect efficacy include age,^24^ sex-concordance,^61^ similarity or dissimilarity of taxa present in either microbiome at the enterotype^62^ or strain level, as well as overall similarities in host immunophenotype. In addition, recipient factors likely strongly impact engraftment in IBD.^28^ Figure 1 summarizes different interacting factors that likely drive clinical improvement following MTT.

*Microbiota compartment transferred may include and not be limited to bacteria, archaea, yeast and other fungi, spores, host virus, and phage

### Recipient preconditioning

Pre-conditioning of the recipient may impact engraftment and possibly clinical outcomes. The use of MTT in rCDI colitis typically follows prolonged antibiotic courses, which dramatically reduce the indigenous microbiota and allows for relatively simple repopulation with donor microbiota. However, while patients with IBD have underlying dysbiosis, the existing established microbiota may need to be disrupted prior to MTT. This can be performed with antibiotics^12,17,23,63^ or bowel lavage. Several trials have used antibiotic pre-treatment as a method to increase engraftment in participants. Regimens given prior to MTT include a combination of: ciprofloxacin and metronidazole for 7 d^12^; amoxicillin, metronidazole, and doxycycline for 2 weeks^17^; amoxicillin, tetracycline, and metronidazole^63^ (to deplete *Fusobacterium varium*, which may contribute to UC pathogenesis),^64^ and amoxicillin, fosfomycin and metronidazole given to UC participants.^23^ One trial that used antibiotic pre-treatment with ciprofloxacin and metronidazole for 7 d followed by an index colonoscopy and 12 weeks of encapsulated MTT found statistically higher donor similarity at the beta community level for MTT versus placebo.^12^ This trial did not have an arm without antibiotics, so it is difficult to tell if antibiotics versus MTT resulted in higher donor similarity. Alternatively, increasing gastrointestinal exposure to MTT can be accomplished by slowing gastrointestinal transit using agents such as loperamide^16,32,65^ or scopolamine.^23^ Also, repeat dosing may be sufficient to overcome the indigenous microbiota produce a change in the recipient microbiota.

Microbiome metanalyses by Podlesny et al. in 2022 for MTT trials for multiple clinical indications (including IBD) found that pre-MTT antibiotics and colonoscopy lavage independently increase engraftment of donor strains.^66^ However, a recent systematic review and metaanalysis of RCTs in UC found that pre-MTT antibiotics and pre-MTT bowel lavage, independent of each other, do not significantly increase combined clinical and endoscopic remission rates in active UC,^39^ and as discussed further below in this review, engraftment may not be the desired outcome of MTT for clinical improvement.

### Host characteristics impact microbiota

Host genetics^21,22^ help determine which intestinal microbiota are present. In IBD, it is well-established that multiple germline mutations affect microbial sensing that may impact tolerance versus inflammation. A key example is the *NOD2* gene, encoding nucleotide-binding oligomerization domain-containing protein 2 that helps sense muramyl peptide components of gram positive and negative bacteria.^67^ Individuals with mutations in *NOD2* risk alleles for developing CD have anywhere from a two to fourfold increased risk (if any one of the main three CD-associated risk alleles are mutated) and up to a 15–40-fold increased risk of developing CD in carriers of two or more of the same affected *NOD2* variants.^68^ Other examples exist such as the protein, Dectin-1, a C-type lectin receptor that functions as a pattern-recognition receptor to affect host responses to fungi^69^ and is implicated in medically refractory UC,^67^ and the gene *FUT2* (that encodes the enzyme fucosyltransferase 2)^70^ that impacts microbiome energy metabolism and risk with CD.^70^ Certain host characteristics may therefore determine the role of MTT in ameliorating intestinal inflammation. Additionally, shared or disparate donor and recipient genetic makeups could in theory impact MTT success. In this sense, lack of donor microbiota engraftment could be akin to a transplanted organ rejection mediated by the recipient’s immune response.

### Why is characterizing engraftment important?

In *C. difficile* colitis, restoration of microbiota may be the mechanism preventing recurrence. Therefore, measures of engraftment may be surrogate markers of key microbial functions that are important in rCDI pathogenesis. In IBD, MTT’s beneficial mechanisms are less established, and therefore the role of engraftment as a surrogate marker for efficacy is unclear. Microbial donor engraftment may aid in reducing inflammation by shifting the immune response to be more favorable toward the new indigenous microbiota. Or engraftment of donor microbiota could fill in missing functional niches, such as secreting local metabolites that promote a functional intestinal epithelial barrier. Due to this, engraftment has become a frequently targeted marker for MTT success in IBD.

### Measuring engraftment

16S rRNA and metagenomic sequencing can be used to measure donor engraftment. Metagenomic sequencing has the potential to identify the mechanism of donor microbiota function. While this can yield information about encoding genes present in microbiota, as well as members present (bacteria, archaea, yeast, fungi, host virus, and phage), including species and strains, it does not yield information about *how* or *whether* genes in the microbiome are expressed. Engraftment can be measured by modeling community beta diversity^71^ or an index combining alpha and beta diversity,^12^ engraftment-trackers,^29,72–74^ or measuring species and strains present in the patient prior versus after MTT and these taxa present in the donor as well as shared taxa. Benefits of using metagenomic data to determine engraftment include resolution to the strain level. 16S rRNA sequencing methods are useful because they often sequence most of what is present in the sample (or have good coverage), but they lack species and strain specificity and functional capacity.

### Does engraftment (or higher donor similarity) translate to improved clinical outcomes in inflammatory bowel disease?

Several clinical trials have shown that MTT increases similarity to donor in IBD patients in UC^12,25,29^ and CD.^15,46^ However, there is wide variability in donor similarity after MTT, and it is unknown whether higher donor similarity is seen in participants who respond clinically in both UC^12,25,29^ and CD.^5,46^ Only two trials in IBD associate clinical response with post-MTT donor similarity (a list of engraftment assessment in blinded RCTs is outlined in Table 1). In the first study by Rossen et al., participants with UC were randomized to MTT or autologous stool transplant.^30^ The composite primary endpoint (a simple clinical colitis activity index ≤2 and ≥1 point improvement on combined endoscopic Mayo score versus baseline at weeks six and 12) was not met, but responders that received healthy donor stool became more similar to their donor (beta community index).^30^ This study also used multiplex PCR probes to sequence the microbiome,^30^ rather than completing 16S rRNA or shotgun metagenomic sequencing, which could limit engraftment assessment.

An open-label, uncontrolled trial by Vaughn et al. also evaluated donor similarity and the relationship to clinical responses in participants with colonic or ileal-colonic CD.^15^ MTT was delivered once via colonoscopy, and 11 of 19 (58%) subjects responded clinically (reduction in Harvey-Bradshaw Index >3) to MTT with 53% of subjects in clinical remission (Harvey-Bradshaw Index <5) at week 4.^15^ Responders experienced a significant change toward donor similarity from baseline as measured by the Bray-Curtis similarity index versus non-responders.^15^ However, the study was small and uncontrolled, limiting any broader conclusions.

Other studies have not clearly identified a trend toward donor microbiota engraftment and clinical outcomes. A study of pediatric CD patients receiving MTT (parental donor) via nasogastric tube did not find a significant association between donor engraftment and clinical response,^46^ which was limited by a small sample size of nine participants. Sokol et al. evaluated MTT in CD participants and did not meet the primary endpoint or donor engraftment as measured by the Sorenson index^5^; however, re-analysis of this trial using metagenomic sequencing found that at the strain level, several participant strains are replaced by donor strains, and that the donor strain haplotype shifted from participant to donor following MTT.^75^ There was also evidence of long-term strain co-existence up to 24 weeks in some, with strains from both the baseline pre-MTT and donor co-existing simultaneously.^75^ However, associations between donor similarity and clinical response were not highlighted.

Several other studies of participants undergoing MTT do not find a correlation of higher donor similarity and clinical response. Schmidt and Li et al. performed a meta-analysis of metagenomic samples from 316 MTT studies for a variety of clinical indications, including rCDI, UC, and CD, and found that both recipient strain displacement and donor strain colonization did not correlate to clinical benefit for any indication,^28^ suggesting that engraftment does not impart clinical improvement. They also did not find any significant differences in strain-level outcomes between non-responders and responders to MTT.^28^ Another meta-analysis by Ianiro et al. of 226 MTT participants for various clinical indications (including IBD and rCDI) investigated strain engraftment and clinical responses and found conflicting results regarding whether engraftment correlated to clinical improvement depending on the statistical test used.^76^ Ianiro et al. stated that their results *suggest* that higher donor similarity *might* improve clinical success of MTT.^76^ Larger meta-analyses of metagenomic data from MTT trials and better data availability are needed to unlock MTT’s mechanisms. But overwhelmingly, current data in the field do not support that engraftment of donor microbiota and strain colonization during MTT improve clinical treatment success in IBD.

## Microbiome post-microbiota transplant therapy

### Taxonomic changes associated with clinical responses

A variety of taxonomic changes post-MTT are associated with clinical outcomes, although these associations are limited by small sample sizes, varying baseline recipient characteristics, donor differences, or MTT route. At baseline, UC patients have higher levels of Bacteroidetes and lower levels of *Clostridium* cluster XIVa.^77^ Increases in *Clostridium* clusters,^6,30^ *Roseburia inulivorans*, ^19^ *Eubacterium hallii*, ^19^ and *Oscillibacter* ^78^ are associated with response post-MTT. However, not all taxonomic associations are consistent. For example, one study with a *Prevotella*-enriched donor found a higher relative abundance of *Prevotella* in recipients to be associated with clinical response.^37^ However, the opposite was found in a different trial in UC recipients given oral, lyophilized donor material that was screened to exclude *Sutterella* and *Fusobacterium;* the *Prevotella*-enriched donor used in this trial was less successful than the other donor with Bacteroides dominance.^17^ The divergence of results from these two trials could be due to route: via colonoscopy in the trial that *Prevotella*-dominant donors were successful^37^ versus oral lyophilized MTT in the trial that *Prevotella*-dominant donors were less successful.^17^

Metagenomic re-analysis by Kong et al.^75^ of the Sokol et al. trial in CD participants^5^ found that engraftment of Bacteroidetes and Proteobacteria were associated with likelihood of relapse, and loss of participant baseline Proteobacteria (*Sutterella wadsworthensis*, *Haemophilus parainfluenzae*, and *Escherichia coli*) occurred in those that did not relapse, suggesting that these species negatively impact CD.^75^ Meta-Cyc pathways associated with relapse included lower normalized pathway abundances in anaerobic energy metabolism, tRNA charging, and NAD biosynthesis I.^75^ In this study, engraftment of *Faecalibacterium prausnitzii* (a butyrate producer) in one patient was not associated with beneficial outcomes,^75^ supporting that transferring SCFA genetic potential may not benefit patients.^28^ As several studies have found different taxa that correlate to clinical responses, and these findings may be tied to either baseline recipient characteristics or donor-recipient compatibility; pooling metagenomic data from multiple trials will be needed to identify whether specific taxa are key to responses.

### Microbial metabolic pathways associated with clinical responses

Elucidating beneficial MTT mechanisms will aid in developing targeted microbiota drug strategies that promote clinical benefit. Two of the most investigated microbial metabolite pathways are those that produce SCFAs and bile acids (BAs). Both SCFAs and BAs affect regulatory T cell and Th17 cells,^79–82^ improve gut barrier function,^83^ and regulate host metabolic pathways.^84^ Trials have either tried to target donors with higher stool levels of SCFAs^12^ or donors that elicit production of SCFAs and T regulatory cells in germ-free mice in response to transfer of human stool^26^; despite this, no improved effects from these donors were seen compared to participants receiving control.^26^ One trial that transferred anaerobic stool (which should help increase viability of bacteria that produce these compounds) did not detect differences in SCFAs in treatment groups or find an association of SCFAs with treatment effect.^16^ One large study that evaluated 1,492 stool metagenomes from participants in MTT trials did not find any correlation of clinical benefit and transfer of specific microbiome functions (such as SCFA production), and donors with genes related to SCFAs did not result in higher strain colonization in recipients.^28^ One MTT trial in UC participants found that responders had increased stool SCFAs following MTT compared to the patients’ baseline levels; however, SCFA levels were not evaluated in non-responders.^85^ MTT trials that have performed analysis of either metagenomic pathways or performed untargeted metabolomics have seen altered pathways for SCFAs and BAs; however, other pathways are often more significantly associated with MTT, suggesting that we should consider other metabolic pathways for mechanisms.

Numerous other microbial-mediated metabolic pathways exist beyond SCFAs and BAs that could account for the clinical benefit from MTT. An untargeted metagenomic analysis of 44 participants with UC undergoing MTT found the top two pathways associated with clinical response were the vitamin B6 and D-glutamine/D-glutamate metabolism.^86^ Vitamin B6 has anti-inflammatory effects on lipopolysaccharide-induced monocytes/macrophages and inhibits NLRP3 inflammasome activation.^87^ Most importantly for IBD, vitamin B6 is required for the degradation of sphingosine-1-phosphate in the colon, which is a potent leukocyte chemoattractant and the drug target of ozanimod.^88^ D-glutamine is important for regulating bacterial division, production of peptidoglycan, spore germination, and regulation of biofilms, both for inhibiting formation^89^ and promoting disassembly.^90^

D-amino acids (which include D-glutamine/glutamate) also have direct immune effects including inhibiting beta-defensin production of epithelial cells (that may alter the host-response to bacteria),^91^ as well as modulating IgA (through regulation of plasma cell numbers in the gut^92^ and regulation of symbiotic bacteria that promote B cell diversification and IgA-class switching).^92^ IgA bound to bacterial surfaces may limit their detection by immune cells in IBD individuals (known as immune-exclusion) to reduce an inflammatory, pathogen-driven immune response against microbiota. Glycosylation sites on IgA also serve as a nutrient source for gut microbiota.^93,94^ One trial evaluating MTT in UC^12^ noted that IgA-coating was higher for bacterial strains transferred from donors (not present in recipients prior to transplant) than for bacterial strains that were present in both the donor and recipient prior to transplant,^95^ indicating that IgA status may drive MTT responses.

## Mycobiome in inflammatory bowel disease undergoing microbiota transplant therapy

The fungal microbiome, or mycobiome, has been evaluated in a few MTT trials in UC participants.^6,30,96,97^ Analysis from both trials in UC participants^6,30^ found that baseline samples with higher *Candida* species relative abundance were associated with clinical response to MTT,^96,97^ and for one trial, this occurred irrespective of the source of MTT (either healthy donor or autologous).^30,97^ Post-MTT, a reduction in *Candida* abundance correlated to a reduction in endoscopic disease severity and clinical disease via the Mayo score.^96^ Functional attributes of *Candida* could contribute to development of inflammation or dysbiosis, as a reduction in *Candida* improves clinical disease. Supporting this, anti-*Candida albicans* IgG in MTT recipients remained stable; however, anti-*C. albicans* IgG in placebo patients increased during the course of the trial.^96^ A reduction in IgG may not have been observed during the course of the trial due to IgG’s long half-life. These results suggest an inflammatory response against *Candida* in UC, inferring that higher baseline levels of *Candida* promote disease. In another trial by van Thiel et al., the abundance of the yeast genus *Filobasidium* in the donor stool correlated to clinical remission (simple clinical colitis activity index ≤2)^97^ following MTT, regardless of the donor source (either healthy or autologous).^30^ Selected species from the *Filobasidium* genus stimulate the release of interleukin-10,^97^ which has been shown to be anti-inflammatory in a murine IBD model.^98^

## Conclusion and future directions

Other than using whole MTT (which increases the risk of infection and adverse events), future microbiota-targeted therapies in IBD may focus on transferring specific bacteria (such as consortia of bacteria that are known to produce metabolites to benefit the host or that stimulate a tolerogenic response from the host immune system), spores,^99^ fungi, or therapies that target bacterial pathobionts, such as bacteriophage^100^ or mycoviruses that target pathogenic fungi. Other potential therapies include a sterile filtrate of MTT^101^ that could contain agents of the microbiome (lipopolysaccharide, DNA, bacterial enzymes) that impart a favorable immune response or specific viruses that target the host immune system or microbiota. In the future, microbiome therapies could be tailored to target specific mutations or polymorphisms in genes involved in immune sensing of the microbiome, as genetic differences may influence individual responses to MTT. It is also possible that healthy, donor-derived MTT might not be necessary; components of autologous (self-derived) MTT could potentially induce a tolerogenic immune response or shape the host microbiome to manage IBD. Such approaches could reduce reliance on broad-spectrum anti-inflammatory medications or the requirement of healthy donors, paving the way for safer, targeted treatment options.

## Data Availability

This is a narrative review. No original data was included.
